# The association between maternal stress and human milk concentrations of cortisol and prolactin

**DOI:** 10.1038/s41598-024-75307-2

**Published:** 2024-11-15

**Authors:** Maja Matyas, Anna Apanasewicz, Małgorzata Krzystek-Korpacka, Natalia Jamrozik, Agnieszka Cierniak, Magdalena Babiszewska-Aksamit, Anna Ziomkiewicz

**Affiliations:** 1 Laboratory of Anthropology, Institute of Zoology and Biomedical Research, Jagiellonian University, Gronostajowa Street 9, Krakow, 30-387 Poland; 2 Department of Anthropology, Hirszfeld Institute of Immunology and Experimental Therapy, Polish Academy of Sciences, 12 Weigla Street, Wroclaw, 53-114 Poland; 3 Department of Medical Biochemistry, Wroclaw Medical University, 10 Chalubinskiego Street, Wroclaw, 50-368 Poland; 4 Faculty of Medicine and Health Sciences, Andrzej Frycz Modrzewski Krakow University, Gustawa Herlinga‑Grudzinskiego 1, Krakow, 30‑705 Poland; 5 Department of Medical Biology, Medical University of Warsaw, Litewska Street 14/16, Warsaw, 00-575 Poland

**Keywords:** Maternal stress, Prolactin, Cortisol, Human milk, Infant development, Gonadal hormones, Steroid hormones, Stress and resilience

## Abstract

Psychosocial stress affects the relationship between prolactin (PRL) and cortisol (CORT). The dynamics of PRL and CORT changes under stress in human milk (HM) are largely unknown. We investigated how maternal stress related to recent life changes affects milk CORT and PRL concentrations. The study involved 116 mothers exclusively breastfeeding 5-month-old infants. Maternal psychological stress was evaluated using the Recent Life Changes Questionnaire (RLCQ). Stress response was determined by administering the cold pressor test and measuring CORT in saliva taken during and in milk collected after the test. Hormones concentrations were assayed using the ELISA method. The hierarchical regression models were run to test the association between maternal RLCQ, salivary CORT, and PRL, and CORT in milk. Maternal RLCQ correlated positively with the CORT in saliva, however, no direct association was found between RLCQ and PRL. After controlling for covariates, a positive association was found between salivary and milk CORT. A negative relationship was observed between salivary CORT and milk PRL. The results of the present study indicate that maternal psychological stress may affect the relationship between CORT and PRL in HM. In response to psychological stress, both hormones transported via milk can program infant development in the early postnatal period.

## Introduction

Current knowledge demonstrates that maternal stress before, during, and after pregnancy can program the future development of the child^1–3^. Studies showed that increased maternal prenatal stress is associated with child brain development, infant cognition and temperamental variation and may be a risk factor for psychopathology in later life^4–7^. In addition, chronic maternal stress during pregnancy associated with stressful life events changes growth trajectories and may predispose to the development of overweight and obesity later in life^8^. It is hypothesized that during the postpartum period, the physiological mechanisms of stress programming rely on the hormonal transmission of signals from the mother to a child via human milk (HM). However, there is also evidence of the impact of maternal stress on child development, whether or not the child is fed HM^9^. For instance, mothers with higher stress, a history of depression and low social support were found to have impaired mother-infant bonding, which may affect infant development^10^. Additionally, studies show that both maternal and paternal depressive symptom are risk factors for later child behaviour problems^11^.

In response to a stressor, the hypothalamic-pituitary-adrenal (HPA) axis is activated, which triggers the cascade of hormonal response, including secretion of corticotropin (CRH), adrenocorticotropin (ACTH) from the brain and glucocorticoids (GC) from the adrenals (1). In addition to their involvement in the stress response, GCs (cortisol, cortisone, and corticosterone) have several essential biological functions. They regulate the metabolism of carbohydrates, proteins, and fats and improve gut maturation, microbiome growth, body composition, and neurodevelopment^12–16^.

HM constitutes a physiological pathway for nutrient transfer and glucocorticoid signalling, which can influence the growth and behaviour of offspring^14,17^. Due to their lipophilic properties, GCs can cross the mammary gland epithelium by simple diffusion^18^, and the concentration of GCs in HM mirrors its concentration in serum. This indicates that GCs may play an important role in transmitting biochemical signals of environmental conditions from mother to infant^19^.

Cortisol (CORT) plays a crucial role in maternal energy metabolism, including gluconeogenesis and lipolysis, and, therefore, has the potential to affect the composition of HM^20,21^ Human and animal research has also evidenced a significant effect of milk CORT on offspring development. Increased concentrations of CORT in milk can be associated with changes in infant behavior^19^, an increase in nervous temperament^14^, and reactivity to stressors^22^. Breastfed infants’ negative early behaviours, such as increased fear and sadness, have been associated with higher CORT concentrations in milk and saliva^2^. Furthermore, a recent human study by Mohd Shukri et al.^23^ indicated improved infant growth in response to reduced stress and lower CORT in HM and maternal saliva. It is also known that maternal traumatic stress is significantly associated with offspring size in the first year of life^24^, which may also be related to altered CORT levels during pregnancy and/or lactation.

Prolactin (PRL) is yet another vital hormone engaged in stress response. Secreted mainly from the pituitary, PRL is a polypeptide hormone inhibiting the HPA axis. This axis can be regulated by PRL from two sources: a synthesis in the anterior pituitary by lactotrophs and release into the peripheral circulation or a synthesis directly in the hypothalamus^25^. Studies on humans showed that PRL increases in response to psychological stressors, including simulated job interviews and mental arithmetic tasks in front of a committee and camera^26^.

Similarly to GCs, PRL plays multiple roles in regulating human metabolism^27^. PRL receptor expression levels increase dramatically during pregnancy and lactation, indicating its essential role in these periods^28^. Evidence connects low PRL concentrations with recurrent miscarriages in women, suggesting that PRL plays a vital role in maternal–fetal interactions during early pregnancy^29^. PRL also plays a key role in initiating maternal behaviour and exerts anxiolytic effects in lactating and non-lactating rats^30^. The study by Gustafson et al. showed that acute exposure to restraint stress increases circulating levels of both prolactin and corticosterone in female mice, and that responses are attenuated during lactation^31^. Studies on rodent models of chronic stress have shown that extended periods of chronic stress significantly decrease plasma prolactin (PRL) levels^32,33^. In a human study by Jergović et al. individuals with chronic PTSD had lower serum PRL concentrations^34^. In contrast, Asher et al.^46^ noted an association between high plasma PRL levels and low anxiety scores in late pregnancy and postpartum, which suggests that, in this case, increased level of PRL associated with lactation may lead to reduced anxiety in lactating women.

Animal studies have shown that PRL is also present in milk and is absorbed intact from the neonatal gut^27,35^. Studies in humans have revealed that the early transmission of PRL from the foremilk to a newborn has an obligatory effect on intestinal fluid and electrolyte exchange^36^. Reduced plasma concentrations of immunoreactive PRL have been linked to intestinal immaturity^37^. It is also likely that PRL in milk acts as a developmental regulator in the gastrointestinal tract and respiratory system^38^. Studies also have confirmed that PRL in HM modulates the development of the acquired immune system in a newborn^38^. However, the overall function of PRL and the factors affecting its level in HM are not entirely understood.

In non-lactating and non-pregnant females, CORT and PRL remain in a dynamic relationship, which depends on the current level of environmental challenge^27^. The increasing level of stress increases CORT secretion. The stress response also includes the release of PRL^39^. However, the dynamics of changes in PRL and CORT concentrations in HM during lactation under the influence of stress are largely unknown.

This exploratory study aims to fill this gap by examining how maternal long-term stress related to recent life changes and salivary cortisol levels affects milk CORT and PRL concentrations in exclusively breastfeeding women. After a sudden, acute stress, HPA axis hormones quickly return to their pre-stress level, but if the stress is repeated or prolonged, it becomes detrimental. Long-term stress dysregulates typical hormone response patterns by causing continuous hormone secretion, which affects metabolism and contributes to mental and physical health disorders^40^. Thus, we hypothesized that maternal stress during lactation and higher maternal CORT response will result in changes in PRL and CORT secretion in HM.

## Methods

### Study group

The study on the association between maternal stress and HM composition involved 160 mothers with their healthy infants from Wroclaw, South-Western Poland. Volunteers were sought through advertisements in local gynaecological clinics, social media (Facebook groups for breastfeeding mothers), radio broadcasts, and newspapers. Recruited participants met the following criteria: for mothers – (a) maternal age at least 18 years old; (b) absence of metabolic diseases such as diabetes or thyroid diseases; (d) exclusively breastfeeding; (e) not drinking alcohol and not smoking cigarettes during pregnancy and lactation; (f) not taking any drugs and steroid treatment including hormonal contraception during pregnancy and lactation; for infants—(a) born on time (at least 37 weeks of gestation) from a single and uncomplicated pregnancy; (b) with birth weight appropriate for gestational age (not lower than 2,500 g); (c) age between 4 and 5 months old; (d) free from any congenital defects, that could affect breastfeeding and infant development.

Complete data on hormone concentrations in milk and saliva were available for 116 women. These women did not differ significantly from the main study group with respect to the basic characteristics such as age, education, socioeconomic status or maternal BMI.

### Study protocol

Mothers and infants were appointed to meet study assistants twice during the study period. The first meeting took place when the children were about five months old. During the meeting, a trained study assistant took anthropometric measurements of the mothers and handed two questionnaires to be completed at home: (1) a general questionnaire collecting basic maternal demographic information such as age, education level (on a 6-level scale from 1–primary to 6–academic; at least a bachelor’s degree), life and economic satisfaction (with a 7-point Likert scale from 1–very unsatisfied to 7–very satisfied), marital status, maternal reproductive history and infant and maternal health; (2) polish version of the Recent Life Changes Questionnaire (RLCQ)^41^ collecting information about maternal stressors. The second meeting occurred one week later when the infant anthropometric measurements and a single milk sample were collected. During the meeting, a hand cold-pressor test (CPT^42^), was performed with saliva samples taken to measure CORT levels. CORT concentrations were determined to assess maternal hormonal response to mild stressors.

The study protocol was approved by the Bioethical Committee of the Lower Silesian Medical Chamber in Wroclaw (approval identification number 1/NT/2016 from 10.02.2016). All mothers signed an informed consent form for the study participation, and all research methods were performed in accordance with relevant guidelines and regulations and with informed consent from all study participants.

### Maternal stress during postpartum

The RLCQ contains a list of 55 events, such as the death of a family member, job loss, divorce, diseases, childbirth, etc., grouped into the domains of health, work, home, family, and personal. With a few exceptions, such as dental treatment, relocation, slight transgression of the law, loss or damage of personal property, most of the significant life-changing events included in the questionnaire are long-term stress related. In addition to listing the events, mothers were asked to report which events they experienced during the last six months before the study and to evaluate the significance and difficulty in adjusting to each of these events on a scale from 1 to 100^41,43^. Points assigned to each stressor were summarized and used in the following analysis as RLCQ scores.

### Cold Pressor test and salivary samples

**Fig. 1:**
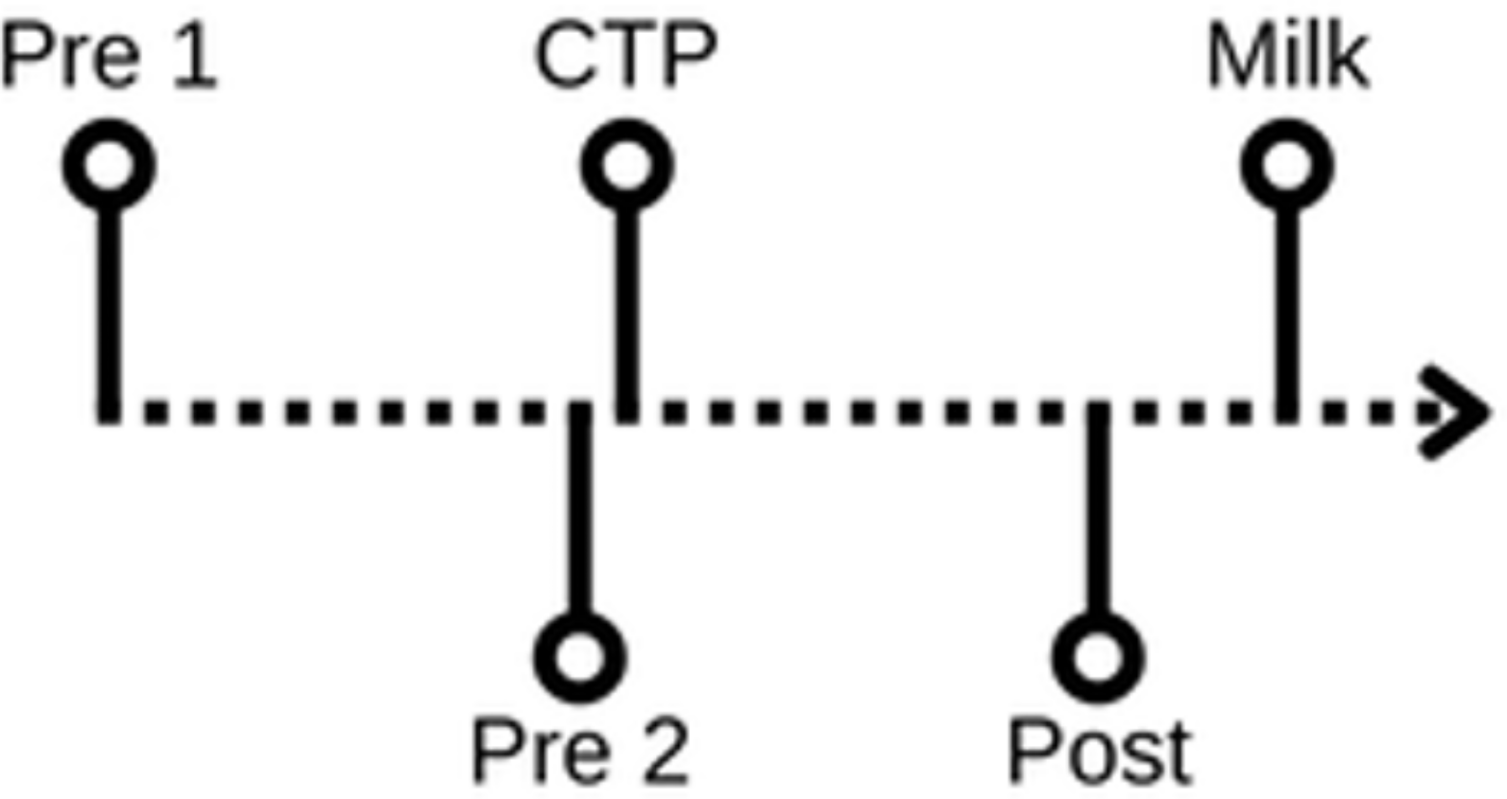
Timeline of sample collection. (Pre 1–1st sample; Pre 2–2nd sample; CTP – 3th sample taken during Cold Pressor Test (CTP); Post – 4th sample; Milk – time of milk sample collection)

To ensure the comfort of breastfeeding mothers, we opted for mild stress stimulation. Women were asked to immerse their hand into a bowl with ice cold water for one minute^44^. None of the participants terminated the test before the expected participation time. Four samples of saliva were taken to measure CORT levels: (1) 10 min and (2) 1 min before, (3) during the test, and (4) 10 min after the test (Fig. 1). The first and second sample was taken pre-test, third sample during the CPT and fourth post-test. Due to diurnal changes in cortisol, tests for all women were conducted between 9 and 11 a.m.

Salivary samples were collected into sterile 1.5 ml Eppendorf tubes and stored at – 80 °C until examination. After thawing and centrifugation (1500 × *g* for 10 min), samples were tested for salivary CORT concentration using enzyme-linked immunosorbent assays (Salivary Cortisol ELISA, DRG Instruments GmbH, Germany) according to the manufacturers’ recommendations. The samples were analyzed in duplicate, and the average intra-assay coefficient of variation for CORT was less than 4.5%.

The average (av) of the collected cortisol concentrations was used for subsequent analyses.

### Milk cortisol

#### Human milk sample collection and analysis

Mothers collected a single milk sample in sterile containers using the Medela Symphony breast pump (Medela AG, Switzerland) under the supervision of the trained research assistant in the study room. Samples were collected no later than 1–1,5 h after the second feeding episode of the day to standardize milk collection time against possible diurnal changes in HM composition^45^. Moreover, the milk samples always were collected after cold pressor test to unify the protocol. Mothers were instructed to pump from one breast until empty. Since research showed no significant difference in milk composition between left and right breast, mothers were free to choose from which breast milk was collected. The minimum amount of milk collected from a mother was 60 ml.

Immediately after collection, milk samples were portioned into smaller containers (1.5, 10, and 15 ml) for further analysis and stored at − 20 °C. Milk aliquots not utilized for immediate analysis were stored at − 80 °C until the time of analysis. CORT was quantified in whole and skim milk. The whole milk samples were centrifuged (12,500 × *g*, 4 °C, 10 min.) to separate cream from skim milk and sediment milk cells. After that, the overlying fat layer was removed, clear milk serum was drawn from the middle of the tube and centrifuged again to achieve skim milk. CORT level was determined by using a Salivary Cortisol Inhibitor ELISA Kit (SLV-4635, DRG Instruments GmbH, Germany) following the protocol published by the producer. CORT concentration was measured calorimetrically at 450 nm using an Infinite M200 plate spectrophotometer (Tecan Group Ltd., Switzerland). Technical replicates were averaged. Free software for curve fitting (available from https://www.mycurvefit.com) and a 4-parameter logistic regression were used for CORT quantification. The samples were analyzed in duplicates, and the average intra-assay coefficient of variation for CORT was less than 14.5%.

### Milk prolactin

Enzyme-Linked Immunosorbent Assay (ELISA) was used to determine the concentration of PRL in milk based on a double-binding assay (Sandwich ELISA) using the Human (PRL) ELISA Kit from Sun Red Biological Technology Co. (China), according to the manufacturer’s instructions. Assay sensitivity was 1.817 ng/ml with 3–600 ng/ml reference standards.

The samples were analyzed in duplicates, and the average intra-assay coefficient of variation for PRL was less than 11.30%.

### Anthropometric measurements

Maternal weight was measured using a Tanita SC-240 MA scale (accuracy 0.1 kg) and height using a stadiometer (accuracy 0.1 cm). These measurements were used to calculate body mass index (BMI). Infant gestational age and size at birth (body weight and length) were taken from the baby’s health record.

### Statistics

Mothers were divided into Low RLCQ and High RLCQ groups according to the median value of the RLCQ score. The low RLCQ group consisted of mothers with RLCQ scores equal to or lower than the median (Mdn = 336.6), while the high RLCQ group consisted of mothers with RLCQ scores higher than the median. Differences in maternal (age, BMI, education, life satisfaction) and infant characteristics (age, body weight, and length), as well as hormone concentrations between these two groups, were tested using the t-test whenever the data indicated normal distribution and the Mann-Whitney U test when the distribution of data diverged from normal.

Data on CORT and PRL was log-transformed (ln) to ensure normality of distribution. Repeated measure analysis of variance (RMANOVA) was used to test the differences in cortisol concentrations between consecutive saliva samples and between low and RLCQ group. Correlation and linear regression were used to test for the effect of recent life stressors and stress on CORT and PRL concentrations. Spearman rank correlation test was applied in the case of RLCQ, which distribution could not be normalized by logarithmic transformation. Two separate hierarchical regression models were run to test for the association between the concentrations of milk PRL and milk CORT and long-term life stressors. In the first step of analysis with milk CORT as a dependent variable, common factors such as maternal BMI, child age, number of feedings, and RLCQ postnatal were included as independent continuous variables in the null model. While in the null model for milk PRL, factors such as BMI, child age, number of feedings, and RLCQ postnatal were included as independent continuous variables. We hypothesized that higher stress led to changes in CORT levels, and CORT caused alterations in milk hormone levels^14,46^; therefore, in the next step, the natural logarithm of salivary CORT level was added in the final model of each analysis. All analyses were conducted using JAMOVI 2.2.5.0.

## Results

The mean mother’s age was 31 years. Their BMI scores ranged from 15.7 to 34.8 kg/m^2^, with an average of 22.7. All infants were born healthy and at term. Their average birth weight was 3,500 g (SD = 0.47), and their length was 54.54 cm (SD = 2.79) (Table 1). Boys constituted 56% of infants. No statistically significant difference except the level of RLCQ was found between groups of mothers with low and high RLCQ. There was also no significant difference between maternal hormone concentrations in low and high RLCQ group except for average salivary cortisol which was higher in high RLCQ group (Table 2).

**Table 1:** Maternal and infant characteristics in all participants, low, and high RLCQ groups.

Characteristics	All (*N* = 116)	Low RLCQ (*N* = 58)	High RLCQ (*N* = 58)
**Maternal**			
Age (years)	31.19 (3.92)	31.40 (4.20)	30.90 (3.66)
BMI (kg/m^2^)	22.73 (3.52)	22.40 (3.16)	23.10 (3.87)
Education^a^	6.00 (0.00)	5.71 (0.77)	5.77 (0.56)
Life Satisfaction ^a^	6.00 (1.00)	5.79 (0.97)	5.86 (0.92)
RLCQ score^a^	365.44 (158.64)	255.75 (105.02)	471.39 (130.42)
**Infant**			
Age (month)	4.76 (0.53)	4.82 (0.59)	4.69 (0.46)
Weight at birth (kg)	3.48 (0.47)	3.49 (0.48)	3.47 (0.46)
Length at birth (cm)	54.54 (2.79)	54.50 (2.89)	54.50 (2.72)

^a^ - Median and interquartile range, RLCQ – Recent Life Changes Questionnaire; unless not stated otherwise, data are presented as means with standard deviation (SD).

**Table 2:** Average value and standard deviation of maternal hormone concentration in all, low, and high RLCQ participants.

Maternal hormones	All	Low RLCQ	High RLCQ
Milk PRL (ng/ml)	29.42 (30.61)	31.6 (30.80)	27.7 (30.60)
Salivary CORT (ng/ml)^av***^	6.74 (7.33)	4.65 (3.97)	8.96 (9.22)
Milk CORT (ng/ml)	4.32 (6.40)	3.78 (5.95)	4.86 (6.90)

Data presented as means with standard deviation (SD). Significant correlations were asterisked. ^***^p < 0.001 RLCQ – Recent Life Changes Questionnaire; PRL – prolactin; CORT – cortisol; av - average.

### Cortisol response to CPT

The level of cortisol was the highest during the pretest (Pre-1) and decreased immediately before the test (Pre-2), during the test (CPT) and 10 min after (Post) (Table 3).

**Table 3:** Average cortisol concentration in each measurement.

Measurement no	Minute of testing	Mean (SD)	Range
Pre 1	0 min	2,49 (2,57)	15,6
Pre 2	10 min	2,33 (2,83)	22,9
CPT	11 min	2,17 (2,52)	19,3
Post	21 min	2,00 (2,47)	18,2

Data presented as means with standard deviation (SD), CPT – Cold Pressor Test.

**Fig. 2:**
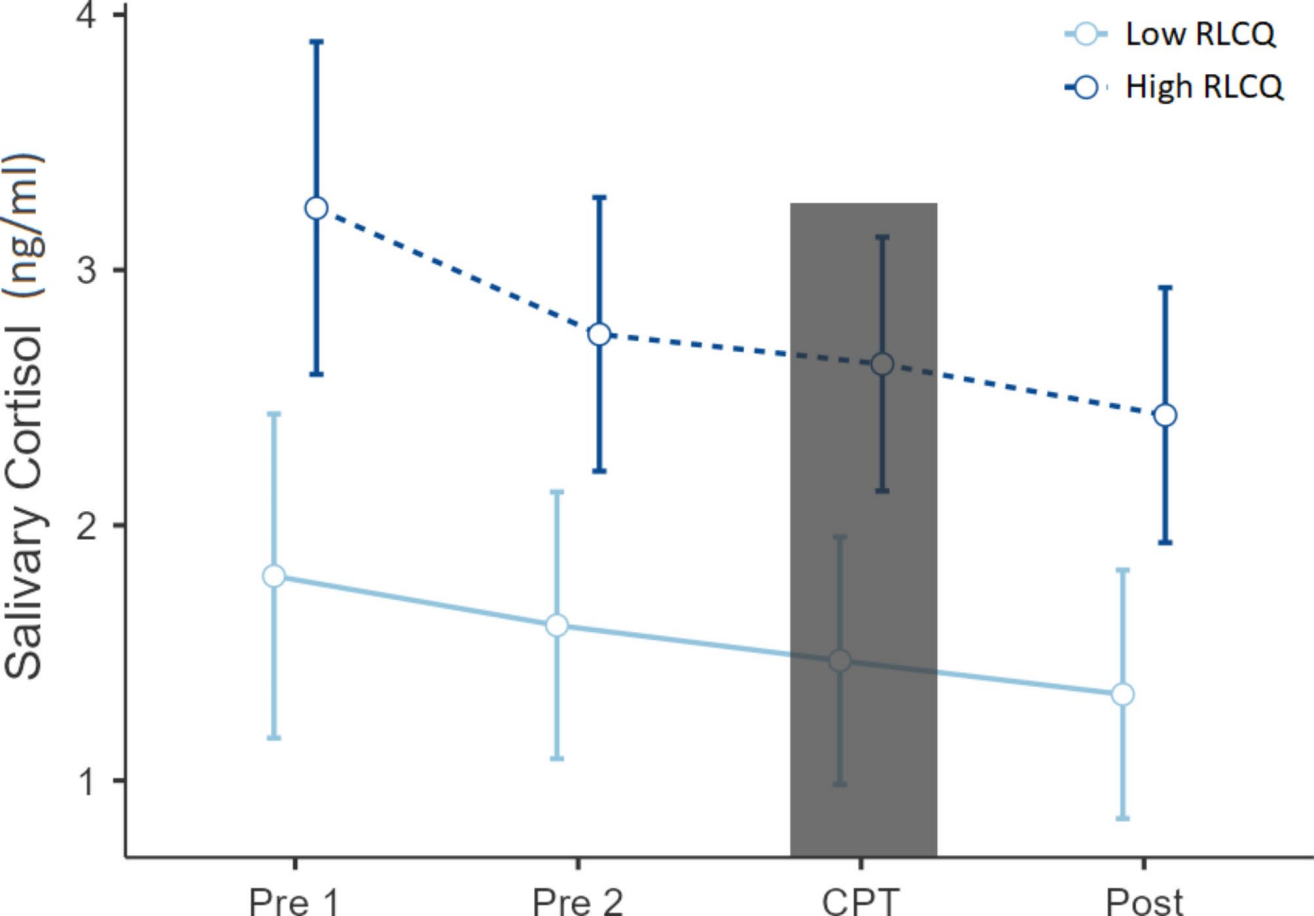
Salivary cortisol at several time points across the experiment. The gray bar represents the time of the stress manipulation. (CPT – Cold Pressor Test; RLCQ – Recent Life Changes Questionnaires)

Changes in cortisol concentrations between the consecutive CPT time points were statistically significant (F_3,230_ = 13.00_;_*p* < 0.001). In particular significant decrease in salivary CORT levels were observed for Pre-1 and all the remaining samples, and for Pre-2 and Post. The average levels of CORT in the consecutive samples taken during the CPT procedure are illustrated at Fig. 2.

In addition, average salivary CORT at consecutive CPT time points significantly differed between study groups of women with low and high RLCQ (F_1,230_ = 11.00; *p* = 0,001).

### Associations between salivary cortisol and milk hormone levels

Significant correlations were found between milk CORT and salivary CORT^av^ (*r* = 0.43; *p* < 0.001). The analysis for potential confounding effects demonstrated a negative association between salivary CORT^av^ and milk PRL (*r* = – 0.21; *p* = 0.022) and between milk PRL and milk CORT (*r* = – 0.25; *p* = 0.005). Salivary CORT^av^ was also correlated with infant age (*r* = – 0.22; *p* = 0.016) and RLCQ (rho = 0.31; *p* < 0.001). In addition, a positive correlation was found between the number of feeding and milk CORT (*r* = 0.18; *p* = 0.049) and between maternal BMI and milk PRL (*r* = 0.24; *p* = 0.008) (Table 4).

**Table 4:** Correlation between maternal hormone concentrations and child age, maternal age, BMI, education, number of feedings, and RLCQ.

	Salivary CORT (ln)^av^	Milk CORT (ln)	Milk PRL (ln)
Child age ^r^	**–0.22** ^*^	–0.07	–0.03
Maternal age ^r^	–0.05	0.07	0.09
Maternal BMI ^r^	–0.02	–0.02	**0.24** ^**^
Maternal education ^r^	0.002	0.12	0.06
Number of feedings ^r^	0.14	0.18	–0.08
RLCQ postnatal ^rho^	**0.31** ^***^	0.14	–0.06

Significant correlations were bolded and asterisked. *p < 0.05; ^**^p < 0.01; ^***^p < 0.001; ln - logarithm.

r – Pearson correlation coefficient; rho – Spearman rank correlation coefficient.

**Fig. 3:**
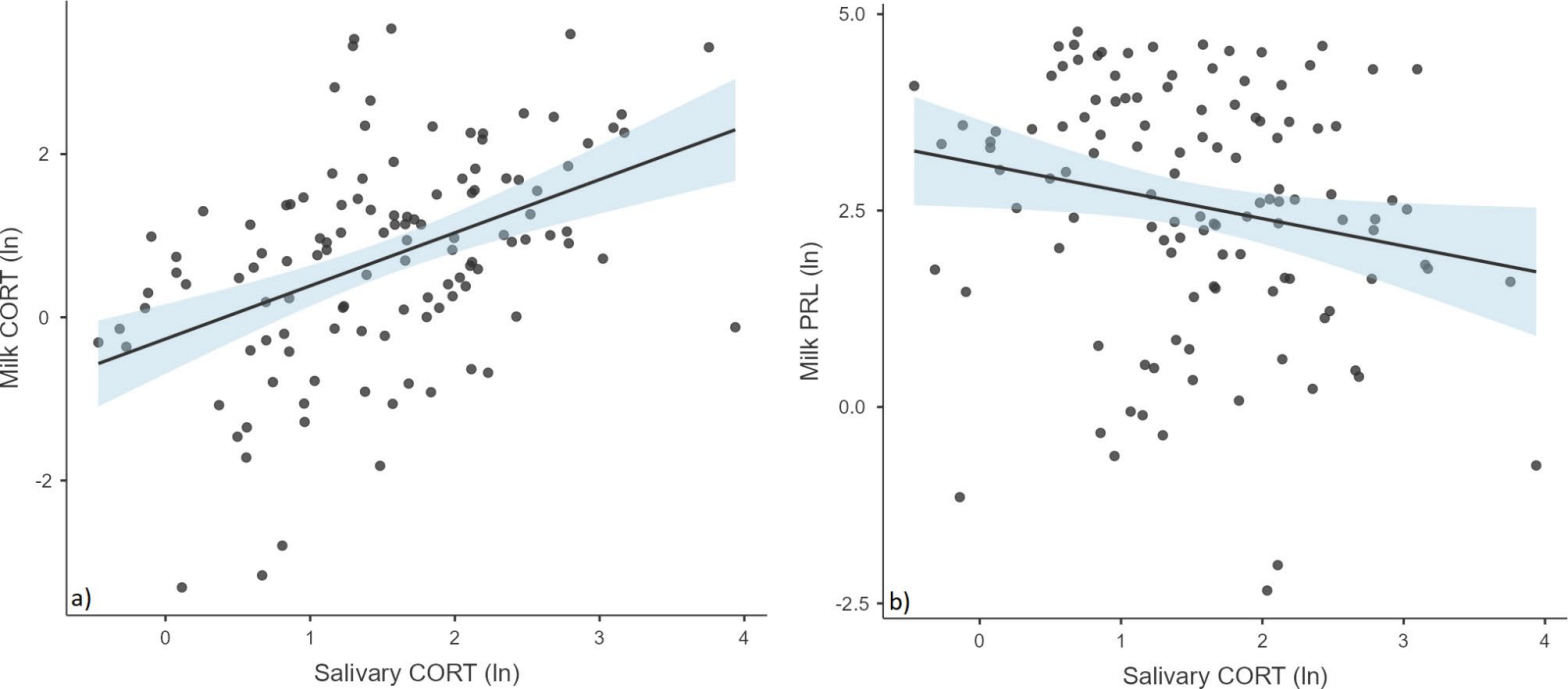
CORT levels in maternal saliva are associated positively with CORT levels (panel **a**) and negatively with PRL levels in HM (panel **b**).

After controlling for covariates in the regression model, a positive association was found between maternal salivary CORT^av^ concentration and milk CORT concentration (β = 0.45; *p* < 0.001) (Fig. 3a). Common factors included in the null model - maternal BMI, child’s age, number of feedings per day, and postnatal RLCQ - together explained about 2% of the variance in the milk CORT concentration. Adding ln salivary CORT^av^ to the model increased the explained variance to 18% (Table 5).

For PRL, a negative relationship was observed between salivary CORT^av^ and milk PRL concentration (β = – 0.24; *p* = 0.017) (Fig. 3b). In the PRL model, common factors included in the null model - maternal BMI, child age, number of feedings per day, and postnatal RLCQ - together explained about 2% of the variance in the milk PRL concentration. Adding ln salivary CORT^av^ increased the variance explained by the model to about 6% (Table 5).

**Table 5:** Results of regression analysis of the association between hormones in milk and saliva. Significant associations were found between milk CORT and saliva CORT and between milk PRL and saliva CORT.

A model with included variables	Adjusted *R*^2^	*p*	*R*^2^ change	*p*	β	95% CI for β	*p*
	**Milk cortisol**						
**Model 0**	0.02	0.201					
Maternal BMI					–0.07	–0.26–0.12	0.474
Child age					–0.11	–0.29–0.08	0.274
Number of feedings					0.18	–0.002–0.37	0.053
RLCQ postnatal					0.09	–0.09–0.28	0.324
**Model 1**	0.18	< 0.001	0.17	< 0.001			
Maternal BMI					–0.05	–0.22–0.12	0.575
Child age					0.02	–0.16–0.20	0.859
Number of feedings					0.11	–0.07–0.28	0.228
RLCQ postnatal					–0.03	–0.20–0.15	0.766
Salivary CORT (ln)					0.45	0.26–0.63	**< 0.001**
	**Milk prolactin**						
**Model 0**	0.03	0.120					
Maternal BMI					0.25	0.06–0.42	**0.009**
Child age					0.02	–0.17–0.21	0.844
Number of feedings					–0.05	–0.24–0.13	0.548
RLCQ postnatal					–0.04	–0.22–0.16	0.677
**Model 1**	0.07	0.021	0.05	0.016			
Maternal BMI					0.24	0.05–0.42	**0.011**
Child age					–0.05	–0.24–0.15	0.626
Number of feedings					–0.01	–0.20–0.17	0.895
RLCQ postnatal					0.03	–0.16–0.22	0.783
Salivary CORT (ln)					–0.24	–0.44–(-0.04)	**0.016**

The significant relations were bolded.

## Discussion

To our knowledge, this is the first study investigating the relationship between maternal psychological stress and PRL concentrations and the association between CORT and PRL concentrations in HM. The results of this study showed that higher response to maternal stress affected by postnatal RLCQ score and indicated by high salivary CORT was associated with elevated concentration of milk CORT and lower milk PRL. Together with our previous research on the effect of stress on milk nutrients, fatty acids^21^, and immunoreactive factors^47^, this study demonstrates a significant role of stress as an essential factor affecting HM composition in women.

### Maternal stress and salivary hormones during lactation

In our study, maternal RLCQ was positively associated with the salivary CORT concentrations during the cold pressor test, which suggests that stress experienced by mothers during postpartum is associated with increased production of CORT in response to a mild laboratory stressor. Mothers with high RLCQ had significantly higher levels of salivary CORT both as average and at the consecutive time points of CPT procedure. Simultaneously, in both low and high RLCQ groups levels of CORT were highest at the pretest and decreased during CPT, which might be related to the anticipatory effect^48^. However, this response was not entirely universal in both group and closer examination of individual CPT CORT profiles indicated significant variance among participants.

It is well-recognized that the amount of CORT secreted in stressful situations correlates with stress intensity^49^. Studies of the association between CORT and RLCQ in the general population show increased CORT secretion under stress^50^. However, this may differ in lactating women^51^. It has been suggested that the HPA axis response to stress is reduced during lactation. That impaired HPA axis response is mediated by the inhibitory effects of oxytocin and PRL released into the blood and specific brain areas^51^. Indeed, a study by Altemus et al.^52^ showed that breastfeeding women have lower plasma ACTH, CORT, and glucose concentration after physical stress compared to non-lactating women. Research by Heinrichs^53^ also confirmed a short-term but significant reduction in maternal psychosocial stress responses during breastfeeding. Similarly, a recent study by Mizuhata et al.^54^ found that breastfeeding significantly reduced maternal salivary CORT levels. Breastfeeding mothers also experienced lower stress levels than mothers using mixed feeding methods. Finally, a study by Meinlschmidt et al.^55^ found no association between psychological stress assessed with the questionnaire and the CORT response in breastfeeding women. The lack of association between stress and CORT may have been influenced by the initiation of breastfeeding by the mothers. Previous studies have shown reduced CORT during breastfeeding^56^ and that breastfeeding women have a lower HPA response to exercise stress than bottle-feeding women^52^. Oxytocin and PRL play a mediating role in this process^57^.

Reduced CORT response to stress may protect breastfeeding mothers, insulating them from adverse environmental factors by reducing the response to stressful stimulation, conserving energy needed for lactation, supporting the immune system of breastfeeding women, and preventing stress-induced lactation inhibition^51^. Reducing CORT response may also benefit the infant by decreasing CORT concentration in the milk. However, no such protective effect was observed in our study, which suggests similar to the general population’s physiological response to long-term stress. Also, the recent study by Zielinska-Pukos et al.^58^ showed no association between milk CORT and maternal or infant factors.

It has to be acknowledged that in contrast to our study, most of the research cited above measured PRL and CORT levels in blood. However, Cadore et al.^59^ showed that salivary hormone levels can be used as a proxy for their respective blood concentrations in the case of CORT. Thus, the increase in salivary CORT concentrations under stress in our study corroborates previous findings based on blood.

### Maternal stress and milk hormones

In contrast to a study by Aparicio and co-authors^60^, we did not find an association between psychosocial stress assessed by RLCQ and the CORT level in HM. Aparicio’s research focused on the relationship between short-term stress and stressful events that occurred up to two months after the study date. Our study was designed to investigate long-term (six months period) maternal stress, so CORT concentrations and their correlations may vary.

Although we did not find the direct effect of perceived stress on milk CORT concentrations, maternal stress response assessed with CORT concentrations was associated with the CORT concentrations in HM. Mothers with higher CORT response had higher concentrations of CORT in HM. These results are in line with previous studies documenting similar positive correlations between maternal plasma and milk CORT concentartionss^61^. They may suggest that CORT is transferred from maternal plasma to milk^62,63^. Bremel & Gangwer’s animal study^61^ confirmed that the mammary gland integrates CORT concentrations in blood. Therefore, based on the results of other studies, it can be assumed that the level of CORT in milk reflects its concentration in plasma. This is supported also by a recent study by Beery et al.^64^, who found a correlation between CORT in milk and CORT in saliva. Further support for the results of our analysis comes from other research on animals. In the study on cattle, serum CORT concentrations increased during psychological stress, leading to increased milk CORT concentrations^65,66^.

Our study also found a negative association between milk and salivary cortisol and prolactin in milk, which may suggest a potential association between maternal long-term stress and milk PRL. The results of studies investigating this relationship are inconclusive, with some showing no change^67,68^, a decrease^34^, or an increase^26^ in PRL concentration in response to stress. Experimental research on chronic stress models in rodents^69,70^ has found that prolonged stress causes a significant reduction in plasma PRL levels. Of all the pituitary hormones, only ACTH and PRL concentrations were reduced after repeated exposure to the same stressor^70^ Another piece of evidence on the adverse effect of stress on PRL comes from research on depression. In a study by Groër^71^, high depression scores were inversely associated with lower serum PRL concentrations. Moreover, the Parotonen study^46^ showed that women with winter depression had low serum PRL levels. In a study by Olff et al.^72^, mean CORT and PRL concentrations were significantly lower in people with post-traumatic stress disorder (PTSD). PTSD is a response to chronic stress and is often associated with HPA axis dysregulation^72^.

In addition, published research has evidenced the positive effect of PRL on stress response. In stressed rats, the involvement of PRL in supporting homeostasis within the immune system has been confirmed, hence the function of PRL as a protective factor in acute stress^73^. Experimentally induced hyperprolactinemia has produced antidepressant effects in rats subjected to forced swimming^74^. Evidence also supports the role of PRL in the adrenal response to stress^39^. PRL can directly induce adrenal steroidogenesis by increasing CORT levels^75^. These observations suggest that CORT and PRL are in coordinated regulation. The negative correlation between salivary CORT and PRL levels in our study may suggest that participants with higher PRL levels produced less CORT in response to stress.

To our knowledge, no other studies have examined the relationship between maternal psychosocial stress and PRL concentrations in animal or human milk. However, PRL concentrations in milk mimic those in plasma^76^. Thus, a similar effect of stress should be expected based on both plasma and milk. In the case of milk PRL, we observed a significant negative correlation with milk CORT, which mimicked the same association found previously in blood. Research suggests that prolactin affects corticotropin-releasing hormone (CRH) neurons in the medial hypothalamic nucleus (PVN), leading to increased cortisol secretion^77^. The correlation between milk PRL and milk and salivary CORT found in our study may suggest a possible link between maternal stress and milk PRL. It also delivers further evidence of the mutual regulation of CORT and PRL not only in serum but also in milk. The physiological role of milk PRL is still poorly understood. Still, animal research suggests that milk PRL plays a role in the offspring’s gastrointestinal^32^ and neuroendocrine^33^ development. Observations from the study by Bermejo-Haro et al.^78^ suggest that milk PRL acts as an immunomodulator and enhances the immune response in the neonatal period.

### Study limitations

One of the possible limitations of our study was the small size of the study group and relatively low variance in the level of health and socioeconomic status. Most of the volunteering mothers had academic education and relatively high socioeconomic status, which could limit the possible number of perceived psychosocial stressors. Thus, some effects could not be detected without the participation of mothers with lower economic status. On the other hand, the homogeneity of the group can also be a benefit by means of limiting the number of confounding factors. All subjects were healthy, so chronic disease, especially disruption of the HPA axis, could not affect the results. In addition, their pregnancies progressed without any severe complications, which could potentially increase the intensity of perceived stress and the level of CORT.

Another limitation may be that we studied CORT concentrations in response to a single and relatively mild stressor. Thus, we do not know how a person behaves and reacts in other, more relevant, stressful situations. To ensure the comfort of breastfeeding mothers, we decided to use mild stress stimulation. Shorter exposure to stressor may have reduced hormone response. However, the cold-pressor test we chose to study is a widely used procedure that activates the sympathetic nervous system with a little effect on cortisol production^79–82^. Usually the measuring points are collected 20 and 30 min after exposition to the stressor. However in the Martins study where samples were collected 10, 20 and 30 min after the CPT, changes in CORT levels were observed as early as 10 min. Therefore, in our study we decided to collect samples during the test and 10 min after the test to limit the amount of time a mother had to spend without her infant.

Finally, it is important to note that the stress reported in the questionnaires does not necessarily reflect physiological responses to the stressors. In the same way, CORT is not only released in a stressful situation, but it can also be released during excitement and joy. This makes difficult to untangle the relationship between the hormones in non-experimental design of the study.

## Conclusion

The results of our study suggest that maternal short-term CORT concentrations to stressors is shaped by perceived long-term stress. Furthermore, these results also show that maternal CORT concentrations are inversely related to the PRL level in milk. This suggests mutual regulation of PRL and CORT. Both hormones regulate human metabolism and are linked to the HPA axis action. When delivered with milk, both can induce adaptive adjustment of infant metabolism to the stressful environment. PRL modulates infant immune function^38,78^ and plays a protective role against the effect of stress^26,74^, which may mitigate the adverse effects of stress on development. Thus, the evidence presented in this paper suggests the possible relevance of CORT–PRL regulation on infant development.

## Data Availability

The data set analyzed during the current study is available from the corresponding author on request.

## Abbreviations

HM: Human milk
HPA: Hypothalamic–pituitary–adrenal
CRH: Corticotropin
ACTH: Adrenocorticotropin
GC: Glucocorticoids
CORT: Cortisol
PRL: Prolactin
RLCQ: Recent Life Changes Questionnaire
CPT: Cold–pressor test
PTSD: Post–traumatic stress disorder

## References

[1] Van Den Bergh, B. R. H., Mulder, E. J. H., Mennes, M. & Glover, V. Antenatal maternal anxiety and stress and the neurobehavioural development of the fetus and child: Links and possible mechanisms. A review. *Neuroscience and Biobehavioral Reviews*, **29**, 237–258 (2005). 10.1016/j.neubiorev.2004.10.007 15811496

[2] Davis, E. P. et al. Prenatal exposure to maternal depression and cortisol influences infant temperament. *J. Am. Acad. Child Adolesc. Psychiatry*** 46**, 737–746 (2007). 17513986 10.1097/chi.0b013e318047b775

[3] Seth, S., Lewis, A. J. & Galbally, M. Perinatal maternal depression and cortisol function in pregnancy and the postpartum period: a systematic literature review. *BMC Pregnancy Childbirth*** 16**, 124 (2016). 10.1186/s12884-016-0915-y PMC4886446 27245670

[4] Lautarescu, A., Craig, M. C. & Glover, V. Prenatal stress: Effects on fetal and child brain development. *Int. Rev. Neurobiol.*** 150**, 17–40 (2020). 32204831 10.1016/bs.irn.2019.11.002

[5] Merced-Nieves, F. M., Dzwilewski, K. L. C., Aguiar, A., Lin, J. & Schantz, S. L. Associations of prenatal maternal stress with measures of cognition in 7.5-month-old infants. *Dev. Psychobiol.*** 63**, 960–972 (2021). 33169388 10.1002/dev.22059 PMC8278565

[6] Lawrence, P. J., Creswell, C., Cooper, P. J. & Murray, L. The role of maternal anxiety disorder subtype, parenting and infant stable temperamental inhibition in child anxiety: a prospective longitudinal study. *J. Child Psychol. Psychiatry*** 61**, 779–788 (2020). 31916250 10.1111/jcpp.13187

[7] Huizink, A. C., De Medina, P. R., Mulder, E. J. H., Visser, G. H. A. & Buitelaar, J. K. Prenatal maternal stress, HPA axis activity, and postnatal infant development. *Int. Congr. Ser.*** 1241**, 65–71 (2002).

[8] Felder, J. N. et al. Prenatal maternal objective and subjective stress exposures and rapid infant weight gain. *J. Pediatr.*** 222**, 45–51 (2020). 32418816 10.1016/j.jpeds.2020.03.017 PMC7731641

[9] Oyetunji, A. & Chandra, P. Postpartum stress and infant outcome: a review of current literature. *Psychiatry Res.*** 284**, 112769 (2020). 31962260 10.1016/j.psychres.2020.112769

[10] Badr, L. K., Ayvazian, N., Lameh, S. & Charafeddine, L. Is the effect of postpartum depression on mother-infant bonding universal?. *Infant Behav. Dev.*** 51**, 15–23 (2018). 29533871 10.1016/j.infbeh.2018.02.003

[11] Carro, M. G., Grant, K. E., Gotlib, I. H. & Compas, B. E. Postpartum depression and child development: an investigation of mothers and fathers as sources of risk and resilience. *Dev. Psychopathol.*** 5**, 567–579 (1993).

[12] Djurhuus, C. B. et al. Additive effects of cortisol and growth hormone on regional and systemic lipolysis in humans. *Am. J. Physiol. Endocrinol. Metab.*** 286**, E488 - E494 (2004). 10.1152/ajpendo.00199.2003 14600073

[13] Reynolds, R. M. Corticosteroid-mediated programming and the pathogenesis of obesity and diabetes. *J. Steroid Biochem. Mol. Biol.*** 122**, 3–9 (2010). 20117209 10.1016/j.jsbmb.2010.01.009

[14] Hinde, K. et al. Cortisol in mother’s milk across lactation reflects maternal life history and predicts infant temperament. *Behav. Ecol.*** 26**, 269–281 (2015). 25713475 10.1093/beheco/aru186 PMC4309982

[15] Cain, D. W. & Cidlowski, J. A. Immune regulation by glucocorticoids. *Nat. Rev. Immunol. *** 17**, 233–247 (2017). 10.1038/nri.2017.1 PMC9761406 28192415

[16] Xu, C., Lee, S. K., Zhang, D. & Frenette, P. S. The Gut microbiome regulates psychological-stress-induced inflammation. *Immunity*** 53**, 417-428.e4 (2020). 32735844 10.1016/j.immuni.2020.06.025 PMC7461158

[17] Dettmer, A. M. et al. Cortisol in neonatal mother’s milk predicts later infant social and cognitive functioning in rhesus monkeys. *Child Dev.*** 89**, 525–538 (2018). 28369689 10.1111/cdev.12783 PMC6528802

[18] Hollanders, J. J., Heijboer, A. C., van der Voorn, B., Rotteveel, J. & Finken, M. J. J. Nutritional programming by glucocorticoids in breast milk: Targets, mechanisms and possible implications. *Best Pract. Res. Clin. Endocrinol. Metab.*** 31**, 397–408 (2017). 29221568 10.1016/j.beem.2017.10.001

[19] Grey, K. R., Davis, E. P., Sandman, C. A. & Glynn, L. M. Human milk cortisol is associated with infant temperament. *Psychoneuroendocrinology*** 38**, 1178–1185 (2013). 23265309 10.1016/j.psyneuen.2012.11.002 PMC4777694

[20] Linderborg, K. M. et al. Interactions between cortisol and lipids in human milk. *Int. Breastfeed. J.*** 15**, 1–11 (2020). 32690057 10.1186/s13006-020-00307-7 PMC7370511

[21] Ziomkiewicz, A. et al. Psychosocial stress and cortisol stress reactivity predict breast milk composition. *Sci. Rep.*** 11**, 11576 (2021). 10.1038/s41598-021-90980-3 PMC8172899 34078999

[22] Nolvi, S. et al. Human milk cortisol concentration predicts experimentally induced infant fear reactivity: moderation by infant sex. *Dev. Sci.*** 21**, (2018). 10.1111/desc.12625 29076272

[23] Mohd Shukri, N. H. et al. Randomized controlled trial investigating the effects of a breastfeeding relaxation intervention on maternal psychological state, breast milk outcomes, and infant behavior and growth. *Am. J. Clin. Nutr.*** 110**, 121–130 (2019). 31161202 10.1093/ajcn/nqz033

[24] Apanasewicz, A. et al. Maternal childhood trauma is associated with offspring body size during the first year of life. *Sci. Rep.*** 12**, 19619 (2022). 10.1038/s41598-022-23740-6 PMC9666509 36380091

[25] Roselli, C. E. et al. Prolactin expression in the sheep brain. *Neuroendocrinology*** 87**, 206–215 (2008). 18223310 10.1159/000114643

[26] Lennartsson, A. K. & Jonsdottir, I. H. Prolactin in response to acute psychosocial stress in healthy men and women. *Psychoneuroendocrinology*** 36**, 1530–1539 (2011). 21621331 10.1016/j.psyneuen.2011.04.007

[27] Torner, L. Actions of prolactin in the brain: From physiological adaptations to stress and neurogenesis to psychopathology. *Front. Endocrinol.*** 7**, 25 (2016). 10.3389/fendo.2016.00025 PMC4811943 27065946

[28] Mitani, S., Amano, I. & Takatsuru, Y. High prolactin concentration during lactation period induced disorders of maternal behavioral in offspring. *Psychoneuroendocrinology*** 88**, 129–135 (2018). 29253704 10.1016/j.psyneuen.2017.12.006

[29] Hanna, C. W., Bretherick, K. L., Liu, C. C., Stephenson, M. D. & Robinson, W. P. Genetic variation within the hypothalamus-pituitary-ovarian axis in women with recurrent miscarriage. *Hum. Reprod.*** 25**, 2664–2671 (2010). 20716560 10.1093/humrep/deq211

[30] Hasiec, M. & Misztal, T. Adaptive modifications of maternal hypothalamic-pituitary-adrenal axis activity during lactation and salsolinol as a new player in this phenomenon. *Int. J. Endocrinol.*** 2018**, 3786038 (2018). 10.1155/2018/3786038 PMC5914094 29849616

[31] Gustafson, P. E. et al. The role of prolactin in the suppression of the response to restraint stress in the lactating mouse. *J. Neuroendocrinol.*** 36**, e13330 (2024) 10.1111/jne.13330 37608555

[32] Akers, R. & Kaplan, R. Role of milk secretion in transport of prolactin from blood into milk. *Horm. Metab. Res.*** 21**, 362–365 (1989). 2777197 10.1055/s-2007-1009238

[33] Grove, D. S., Bour, B., Kacsóh, B. & Mastro, A. M. Effect of neonatal milk-prolactin deprivation on the ontogeny of the immune system of the rat. *Endocr. Regul.*** 25**, 111–119 (1991). 1958825

[34] Jergović, M. et al. Circulating Levels of hormones, lipids, and immune mediators in post-traumatic stress disorder—a 3-month follow-up study. *Front. Psychiatry*** 6**, 49 (2015). 10.3389/fpsyt.2015.00049 PMC4396135 25926799

[35] Tissier, L. R., Hodson, D. J., Martin, A. O., Romanò, N. & Mollard, P. Plasticity of the Prolactin (PRL) axis: mechanisms underlying regulation of output in female mice. *Adv. Exp. Med. Biol.*** 846**, 139–162 (2015). 25472537 10.1007/978-3-319-12114-7_6

[36] Ho Yuen, B. Prolactin in human milk: the influence of nursing and the duration of postpartum lactation. *Am. J. Obstet. Gynecol.*** 158**, 583–586 (1988). 3348320 10.1016/0002-9378(88)90032-4

[37] Ellis, L. A. & Picciano, M. F. *Bioactive and Immunoreactive Prolactin Variants in Human Milk**. *Endocrinology *138, 2711–2720 (1995). 10.1210/endo.136.6.7750496 7750496

[38] Ellis, L. A., Mastro, A. M. & Picciano, M. F. Milk-borne prolactin and neonatal development. *J. Mammary Gland Biol. Neoplasia *1, 259-269 (1996). 10.1007/BF02018079 10887500

[39] Levine, S. & Muneyyirci-Delale, O. Stress-induced hyperprolactinemia. *Pathophysiol. Clin. Approach.*** 2018**, 9253083 (2018). 10.1155/2018/9253083 PMC6304861 30627169

[40] Parker, V. J. & Douglas, A. J. Stress in early pregnancy: maternal neuro-endocrine-immune responses and effects. *J. Reprod. Immunol.*** 85**, 86–92 (2010). 20079933 10.1016/j.jri.2009.10.011

[41] Adam, S., Jan, S. & Bogdan, Z. Kwestionariusz Zmian Życiowych (KZŻ). *Przegląd Psychol.*** 42**, 27–49 (1999).

[42] Seneviratne, B. I. B., Linton, I., Wilkinson, R., Rowe, W. & Spice, M. Cold pressor test in diagnosis of coronary artery disease: echophonocardiographic method. *Br. Med. J.*** 286**, 1924–1926 (1983). 6407637 10.1136/bmj.286.6382.1924 PMC1548288

[43] Rahe, R. H. Epidemiological studies of life change and illness. *Int. J. Psychiatry Med.*** 6**, 133–146 (1975). 773851 10.2190/JGRJ-KUMG-GKKA-HBGE

[44] Martins, V. J. B. et al. Normal cortisol response to cold pressor test, but lower free thyroxine, after recovery from undernutrition. *Br. J. Nutr.*** 115**, 14–23 (2016). 26525425 10.1017/S0007114515004225

[45] van der Voorn, B. et al. Breast-milk cortisol and cortisone concentrations follow the diurnal rhythm of maternal hypothalamus-pituitary-adrenal axis activity. *J. Nutr.*** 146**, 2174–2179 (2016). 27629575 10.3945/jn.116.236349

[46] Partonen, T. *Prolactin in Winter Depression*. *Medical Hypotheses I Medical Hypothrsrs*, **43**, 163-164 (1994). 10.1016/0306-9877(94)90145-7 7815971

[47] Ziomkiewicz, A. et al. Maternal distress and social support are linked to human milk immune properties. *Nutrients*** 13**, 1857 (2021). 10.3390/nu13061857 PMC8226629 34072410

[48] Juster, R.-P., Perna, A., Marin, M.-F., Sindi, S. & Lupien, S. J. Timing is everything: anticipatory stress dynamics among cortisol and blood pressure reactivity and recovery in healthy adults. *Stress*** 15**, 569–577 (2012). 22296506 10.3109/10253890.2012.661494

[49] Hellhammer, D. H., Wüst, S. & Kudielka, B. M. Salivary cortisol as a biomarker in stress research. *Psychoneuroendocrinology*** 34**, 163–171 (2009). 19095358 10.1016/j.psyneuen.2008.10.026

[50] Sussams, R. et al. Psychological stress, cognitive decline and the development of dementia in amnestic mild cognitive impairment. *Sci. Rep.*** 10**, 3618 (2020). 32108148 10.1038/s41598-020-60607-0 PMC7046646

[51] Heinrichs, M., Neumann, I. & Ehlert, U. Lactation and stress: protective effects of breast-feeding in humans. *Stress*** 5**, 195–203 (2002). 12186682 10.1080/1025389021000010530

[52] Altemus, M., Deuster, P. A., Galliven, E., Carter, C. S. & Gold, P. W. Suppression of hypothalmic-pituitary-adrenal axis responses to stress in lactating women. *J. Clin. Endocrinol. Metab.*** 80**, 2954–2959 (1995). 7559880 10.1210/jcem.80.10.7559880

[53] Heinrichs, M. et al. Effects of suckling on hypothalamic-pituitary-adrenal axis responses to psychosocial stress in postpartum lactating women. *J. Clin. Endocrinol. Metab.*** 86**, 4798–4804 (2001). 11600543 10.1210/jcem.86.10.7919

[54] Mizuhata, K., Taniguchi, H., Shimada, M., Hikita, N. & Morokuma, S. Effects of breastfeeding on stress measured by saliva cortisol level and perceived stress. *AsianPacific Isl. Nurs. J.*** 5**, 128–138 (2020). 10.31372/20200503.1100 PMC7733634 33324730

[55] Meinlschmidt, G., Martin, C., Neumann, I. D. & Heinrichs, M. Maternal cortisol in late pregnancy and hypothalamic–pituitary–adrenal reactivity to psychosocial stress postpartum in women. *Stress*** 13**, 163–171 (2010). 20214437 10.3109/10253890903128632

[56] Amico, J. A., Johnston, J. M. & Vagnucci, A. H. Suckling-induced attenuation of plasma cortisol concentrations in postpartum lactating women. *Endocr. Res.*** 20**, 79–87 (1994). 8168464 10.3109/07435809409035858

[57] Grattan, D. R. et al. Prolactin receptors in the brain during pregnancy and lactation: implications for behavior. *Horm. Behav.*** 40**, 115–124 (2001). 11534971 10.1006/hbeh.2001.1698

[58] Zielinska-Pukos, M. A. *et al.* Factors influencing cortisol concentrations in breastmilk and its associations with breastmilk composition and infant development in the first six months of lactation. *Int. J. Environ. Res. Public. Health*** 19**, 14809 (2022). 10.3390/ijerph192214809 PMC9690377 36429527

[59] Cadore, E. et al. Correlations between serum and salivary hormonal concentrations in response to resistance exercise. *J. Sports Sci.*** 26**, 1067–1072 (2008). 18608830 10.1080/02640410801919526

[60] Aparicio, M. et al. Human milk cortisol and immune factors over the first three postnatal months: relations to maternal psychosocial distress. *PLOS One*** 15**, e0233554 (2020). 32437424 10.1371/journal.pone.0233554 PMC7241837

[61] Bremel, R. D. & Gangwer, M. I. Effect of adrenocorticotropin injection and stress on milk cortisol content. *J. Dairy Sci.*** 61**, 1103–1108 (1978). 214474 10.3168/jds.S0022-0302(78)83693-5

[62] Kato, E. A., Hsu, B. R., Raymoure, W. J. & Kuhn, R. W. Evidence for the direct transfer of corticosteroid-binding globulin from plasma to whey in the guinea pig. *Endocrinology*** 117**, 1404–1408 (1985). 3875481 10.1210/endo-117-4-1404

[63] Kulski, J. K. & Hartmann, P. E. Changes in the concentration of cortisol in milk during different stages of human lactation. *Aust. J. Exp. Biol. Med. Sci.*** 59**, 769–778 (1981). 7340774 10.1038/icb.1981.66

[64] Beery, A. K. et al. Acute decrease in mothers’ cortisol following nursing and milk expression. *Horm. Behav.*** 153**, 105387 (2023). 37307679 10.1016/j.yhbeh.2023.105387

[65] Termeulen, S. B., Butler, W. R. & Natzke, R. P. Rapidity of cortisol transfer between blood and milk following adrenocorticotropin injection. *J. Dairy Sci.*** 64**, 2197–2200 (1981). 6278010 10.3168/jds.S0022-0302(81)82829-9

[66] Verkerk, G. A., Phipps, A. M. & Matthews, L. R. Milk cortisol concentrations as an indicator of stress in lactating dairy cows. *Proc. N. Z. Soc. Anim. Prod.*** 56**, 77-79 (1996).

[67] Lacey, K. et al. A prospective study of neuroendocrine and immune alterations associated with the stress of an oral academic examination among graduate students. *Psychoneuroendocrinology*** 25**, 339–356 (2000). 10725611 10.1016/s0306-4530(99)00059-1

[68] Song, Y., Zhou, D. & Wang, X. Increased serum cortisol and growth hormone levels in earthquake survivors with PTSD or subclinical PTSD. *Psychoneuroendocrinology*** 33**, 1155–1159 (2008). 18640782 10.1016/j.psyneuen.2008.05.005

[69] Chen, Y. et al. Sex differences in peripheral monoamine transmitter and related hormone levels in chronic stress mice with a depression-like phenotype. *PeerJ*** 10**, e14014 (2022). 36132219 10.7717/peerj.14014 PMC9484450

[70] Faron-Górecka, A. et al. Prolactin and its receptors in the chronic mild stress rat model of depression. *Brain Res.*** 1555**, 48–59 (2014). 24508286 10.1016/j.brainres.2014.01.031

[71] Groër, M. W. Differences between exclusive breastfeeders, formula-feeders, and controls: a study of stress, mood, and endocrine variables. *Biol. Res. Nurs.*** 7**, 106–117 (2005). 16267372 10.1177/1099800405280936

[72] Olff, M., Güzelcan, Y., de Vries, G.-J., Assies, J. & Gersons, B. P. R. HPA- and HPT-axis alterations in chronic posttraumatic stress disorder. *Psychoneuroendocrinology*** 31**, 1220–1230 (2006). 17081699 10.1016/j.psyneuen.2006.09.003

[73] Ochoa-Amaya, J. E. et al. Acute and chronic stress and the inflammatory response in hyperprolactinemic rats. *Neuroimmunomodulation*** 17**, 386–395 (2010). 20516720 10.1159/000292063

[74] Drago, F., Pulvirenti, L., Spadaro, F. & Pennisi, G. Effects of TRH and prolactin in the behavioral despair (swim) model of depression in rats. *Psychoneuroendocrinology*** 15**, 349–356 (1990). 2129310 10.1016/0306-4530(90)90060-m

[75] Glasow, A. et al. Functional aspects of the effect of prolactin (PRL) on adrenal steroidogenesis and distribution of the PRL receptor in the human adrenal gland. *J. Clin. Endocrinol. Metab.*** 81**, 3103–3111 (1996). 8768882 10.1210/jcem.81.8.8768882

[76] Schams, D. & Karg, H. Hormones in milk. *Ann. N. Y. Acad. Sci.*** 464**, 75–86 (1986). 3524354 10.1111/j.1749-6632.1986.tb15995.x

[77] Faron-Górecka, A. et al. The involvement of prolactin in stress-related disorders. *Int. J. Environ. Res. Public. Health*** 20**, 3257 (2023). 36833950 10.3390/ijerph20043257 PMC9959798

[78] Bermejo-Haro, M. Y., Camacho-Pacheco, R. T., Brito-Pérez, Y. & Mancilla-Herrera, I. The hormonal physiology of immune components in breast milk and their impact on the infant immune response. *Mol. Cell. Endocrinol.*** 572**, 111956 (2023). 37236499 10.1016/j.mce.2023.111956

[79] al’Absi, M., Nakajima, M. & Bruehl, S. Stress and pain: modality-specific opioid mediation of stress-induced analgesia. *J. Neural Transm.*** 128**, 1397–1407 (2021). 34405305 10.1007/s00702-021-02401-4 PMC8664159

[80] Sofowora, G. G., Singh, I., He, H. B., Wood, A. J. J. & Stein, C. M. Effect of acute transdermal estrogen administration on basal, mental stress and cold pressor-induced sympathetic responses in postmenopausal women. *Clin. Auton. Res. Off. J. Clin. Auton. Res. Soc.*** 15**, 193–199 (2005). 10.1007/s10286-005-0261-z 15944868

[81] Wu, T., Snieder, H. & de Geus, E. Genetic influences on cardiovascular stress reactivity. *Neurosci. Biobehav. Rev.*** 35**, 58–68 (2010). 19963006 10.1016/j.neubiorev.2009.12.001

[82] Goodin, B. R., Smith, M. T., Quinn, N. B., King, C. D. & McGuire, L. Poor sleep quality and exaggerated salivary cortisol reactivity to the cold pressor task predict greater acute pain severity in a non-clinical sample. *Biol. Psychol.*** 91**, 36–41 (2012). 22445783 10.1016/j.biopsycho.2012.02.020 PMC3606711

